# Application of human–computer interaction virtual reality technology in urban cultural creative design

**DOI:** 10.1038/s41598-023-41566-8

**Published:** 2023-09-01

**Authors:** Sujuan Han, Shuo Liu, Lili Ren

**Affiliations:** 1 College of Fine Arts and Design, Heze University, Heze, China; 2 Solux College of Architecture and Design, University of South China, Hengyang, China

**Keywords:** Ecology, Environmental social sciences

## Abstract

As the in-depth expansion and integrated application of information technology, smart city is one of the important directions for nurturing breakthroughs in the new generation of information technology, and an important part of the development of global strategic emerging industries. In the context of world peace, the world economy is developing rapidly and the process of urbanization is accelerating. The construction of a city not only reflects the economic strength of the city, but also is closely related to the life of the citizens. Therefore, this paper aimed to explore a new way of urban cultural creative design. This paper focused on the application of human–computer interaction virtual reality (VR) technology in urban cultural creative design. For this reason, this paper designed an interactive and immersive urban design simulation platform based on VR technology. In order to choose the viewing angle freely and control the design module freely, this paper proposed improved gesture recognition algorithm and anti-collision algorithm. The algorithm designed in this paper can enable designers to have better presentation effects when working on the platform, making architectural design more intuitive. Experiments show that the average accuracy rate of the gesture recognition algorithm designed in this paper reaches 97.3%, which is 4.1% higher than that of the traditional algorithm; and when the anti-collision algorithm in this paper is set to the optimal parameters, the accuracy rate is as high as 95%. These results fully demonstrate that the human–computer interaction VR technology design platform proposed in this paper has good design performance and can be applied to urban cultural creative design.

## Introduction

The urban development concept of smart city with Internet of things, cloud computing and other technologies as the core has subverted the traditional thinking that urban physical infrastructure and it information infrastructure are completely separated, effectively linked various facilities in the city, and provided a new value concept for urban development^1^. At present, many cities are experiencing severe challenges such as economic transformation, industrial restructuring, and deterioration of the ecological environment. Cultural revival is an effective way of urban regeneration, which has achieved certain success in other countries. However, the current urban transformation is mainly focused on large-scale commercial development. It pays too much attention to the creation of the material environment and does not fully understand the impact of cultural renaissance on urban society, economy and culture. In this context, this paper believed that the urban cultural transformation in the new era should focus on the existing various cultural resources and cultural spaces. Urban cultural space is an urban element with strong sense of participation, strong recognizability, and concentrated reflection of urban history and culture. Its development and the revival of the city complement each other: the revival of the city is an opportunity for the development of cultural space; and the development of cultural space also provides the necessary factors for the revival of the city. Therefore, this paper took the “urban cultural space” as the research object, and defines it as the “cultural space” at the “community” level from the perspective of the conflict between the current architectural situation and the needs of the people. Then it used the VR technology of human–computer interaction to design an interactive and immersive urban design platform, so that it can be integrated with the local culture and become a more distinctive modern city.

Many scholars have conducted research on urban construction design architecture, sociology, art and other disciplines. Zhang^2^ took micro-history theory as the basis of project development, and explores how to use architecture as a carrier to awaken the urban development of micro-history. Rudwiarti et al.^3^ provided new ideas for urban construction from the perspective of disabled people, and carried out urban construction under the social culture of caring for disabled people. Haque et al.^4^ studied the relationship between urban cultural construction and family smoking ban. He believed that a large urban atmosphere can greatly improve family pressure, so that smoking behavior becomes less. On the basis of analyzing the meaning expressed by the cultural vocabulary of landscape facilities, Dongmei et al.^5^ explained the application of the cultural vocabulary of landscape facilities through practical cases, and then analyzed the modern design of urban architecture. Bogucka et al.^6^ collected a dataset of 5000 streets in cities such as Paris, Vienna, London, and New York, and constructed their cultural maps based on cartographic techniques to summarize new ideas for urban cultural construction. It can be found that urban construction has always been a hot issue of social concern. Relevant scholars have not only analyzed the impact of urban construction on citizens, but also discussed the concept of urban construction from multiple perspectives. However, most of the relevant scholars' research is still in theoretical investigation, lacking discussion on actual urban construction design, and lack of application of high-tech.

Some scholars have also studied the application of VR technology in urban construction. On the basis of summarizing and analyzing previous research work, Liu^7^ expounded the research status and significance of three-dimensional (3D) visualization urban landscape planning and design, and expounded the development background, current situation and future challenges of VR technology. Guo^8^ has demonstrated through practice that using virtual city 3D spatial visualization technology for urban management can optimize urban management and services, improve the efficiency of public services, and enhance the convenience of citizens’ lives. Virtual city 3D spatial visualization has good prospects in urban planning and management, virtual community service management, and other fields. Chen et al.^9^ used virtual reality (VR) and photoplethysmography (PPG) technologies, physiological eye movement and heart rate variability (HRV) data, as well as psychological data obtained from positive and negative emotional adjectives (PANA), to evaluate the subject satisfaction of urban green space plant community landscape scenes. The research results provided theoretical guidance for landscape design based on human perception preferences and the implementation of sustainable landscape in the context of carbon neutrality, to achieve a win–win situation where the benefits of carbon sequestration and oxygen release can coexist with aesthetics. Xu and Zheng^10^ has developed an immersive, interactive multi-person training platform incorporating VR technology to improve workers’ safety awareness in urban construction and stimulate human–computer interaction research. Tabrizian et al.^11^ digitally processed photorealistic 360° panoramas taken from squares and parks to create 18 immersive virtual environment (IVE) stimuli depicting changes in spatial arrangement and vegetation permeability. However, relevant scholars pay more attention to the design of platform functions, and lack the consideration of cultural elements in urban construction, which leads to the separation of urban construction from culture.

Urban planning not only pays attention to the creation of the physical environment, but also stabilizes the community and promotes the inheritance and innovation of urban culture. The planning also optimizes the urban traffic environment, enhances the sense of belonging to the community, and connects with the non-material aspects of the city’s society, economy, culture, etc., which is a necessary means for the comprehensive revival of the city. To this end, this paper attempted to rely on the theory and method of urban design to explore the urban cultural space that is people-oriented, inherits the historical context of the city, and is full of vitality.

## Urban cultural creative design

### Current situation of Chinese urban culture

#### The phenomenon of urban appearance convergence is serious

**Figure 1:**
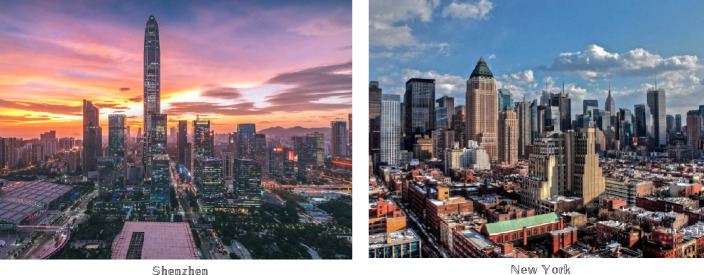
“One Thousand Cities One Side” Phenomenon^13, 14^.

The phenomenon of urban appearance convergence has become a significant issue in recent years. Each city possesses its own unique cultural characteristics, which have been developed and accumulated over various historical stages. However, with the rapid advancement of economic globalization in the twenty-first century, small and medium-sized cities in China have increasingly embraced global culture, leading to a significant impact on their local cultural identity^12^. Consequently, the original urban cultural traits have begun to deteriorate, and the distinctive style of the cities has been overshadowed by the construction of new districts. This has resulted in the emergence of a growing trend known as “a thousand cities is one side,” as depicted in Fig. 1^13, 14^.

#### Insufficient construction of cultural space at the community level

The concept of cultural plans emerged in major western cities during the 1970s, playing a pivotal role in promoting urban development by harnessing a city's cultural resources. A cultural plan is essentially a strategic blueprint that addresses the utilization of cultural resources and fulfills cultural needs. It encompasses various aspects such as the economy, transportation, infrastructure, land use, and historical preservation, with implementation predominantly focused on regional and urban scales.

The successful urban renewal project in Bilbao stands as a global model that showcases two significant outcomes. Firstly, it provides a pathway for improving the overall quality of a city. Secondly, it exemplifies how a city's image transformation can catalyze economic development and attract investments. Consequently, since the beginning of the twenty-first century, numerous large cities in China have experienced a proliferation of “museums.” These cities aim to leverage the construction of large-scale cultural buildings as a means to stimulate economic growth and drive the development of new districts. Consequently, many cities have strategically prioritized the construction of large-scale cultural and art centers, grand theaters, museums, and exhibition centers. This surge has witnessed the emergence of substantial investments, ranging from hundreds of millions to tens of billions. A striking example is the establishment of 395 museums in China in 2010 alone, demonstrating an astounding daily increase in the number of museums^15^.

However, an evaluation of the current state of cultural space construction in China reveals a notable lack of emphasis on cultural space creation within the transitional zone of urban planning and architecture, specifically in urban design. Typically, urban planning tends to focus on the macro form of the city, while architecture concerns itself with the micro form of cultural spaces. Consequently, cultural spaces situated at the community level, which lie in the intermediate zone between the two, are often overlooked.

### Citizens’ contemporary demands for urban cultural space

#### Demands for urban pride and self-identification

As globalization continues to advance, developed cities have adopted various policies, such as the importation of films and television shows and the integration of enterprises, to counter the economic challenges faced by less developed cities. In this context, the cultural industry has emerged as a primary driver of urban development. The cultural value of a city is gradually gaining significance as a key symbol of its progress.

**Figure 2:**
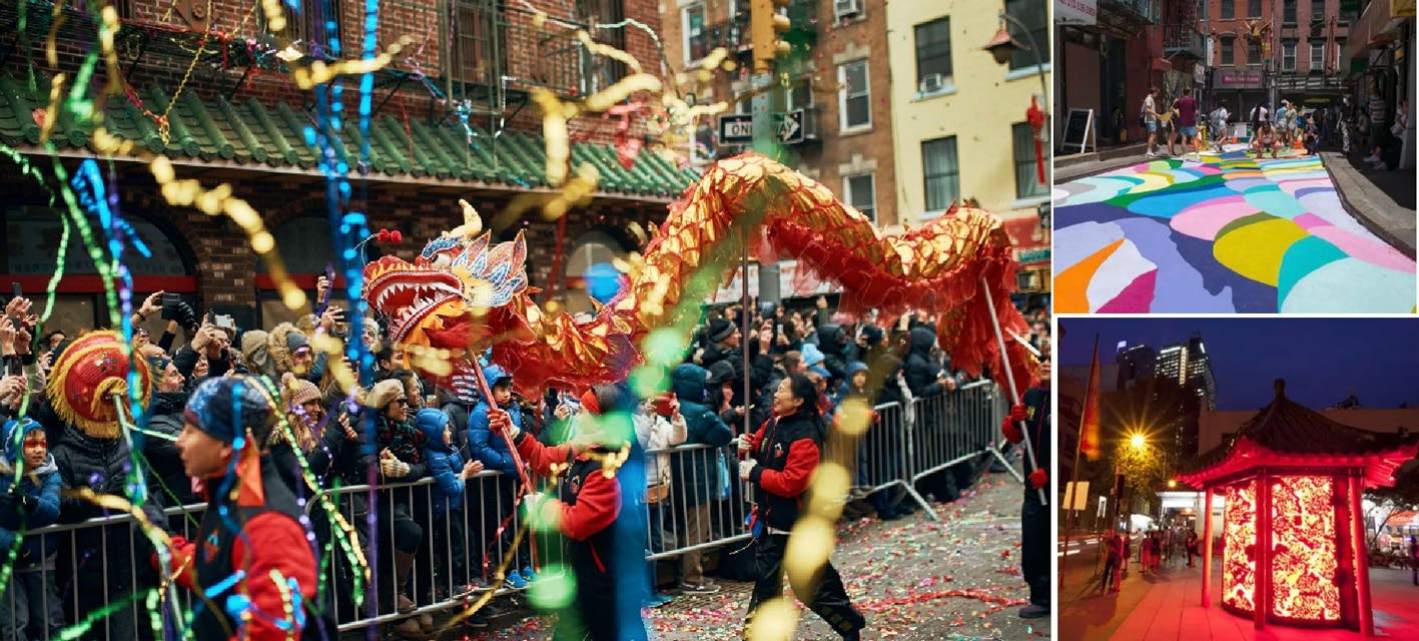
Celebrations in Manhattan’s Chinatown^17^.

At the same time, citizens are also looking for their own cultural identity in the ever-changing city. While feeling the impact of foreign cultures, locals are also eager to carry forward and inherit their own traditions and cultures, thereby enhancing their pride in the city. The development of urban cultural space needs to reflect distinct regional characteristics. In the planning, cultural resources should be fully explored, combined with characteristic resources, involved in characteristic cultural elements, and organized traditional cultural activities^16^. Taking New York's Chinatown as an example, the annual Spring Festival float parade is already a major feature of New York City. In particular, parades, firecrackers, gongs and drums, dragon dances, and lion dances are a major feature of New York during the celebrations held in Manhattan's Chinatown, as shown in Fig. 2.

#### Concerns about cultural industry space under the cultural consumption boom

In the 1940s, the famous scholar Adorno put forward the concept of “cultural industry”. He believes that the development of industrial technology would also change the way culture is created. It goes from literature to automation to mass production and innovation^18^. The product of cultural industry has become a kind of fast spiritual consumer goods that satisfy the public's taste, and the essence of cultural consumption is a kind of control over society. “Cultural industry” is to meet the needs of the masses as the main goal, mass production of products to meet the needs of the masses. The emergence of “cultural industry” destroys traditional humanistic values, making it secular and mediocre. The postmodern view of cultural consumption points out that it is incorrect to regard cultural consumption as a means of social control, but it still recognizes the cultural significance of the cultural industry and believes that it is essentially a cultural creation.

**Figure 3:**
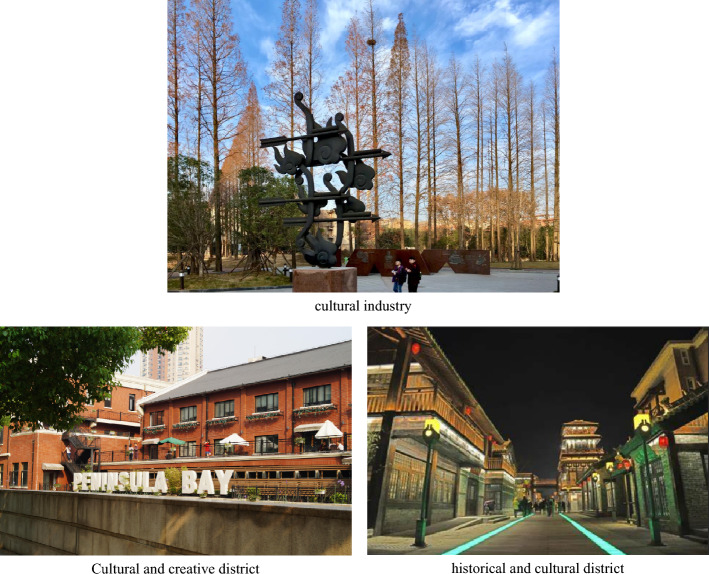
Cultural industry.

The cultural industry has brought about great changes in the cultural connotation. Culture undergoes production, sales and consumption like an ordinary object. With the commercialization of culture, the space for cultural production and dissemination has also received more and more attention in urban construction^19^. Today, when popular culture and consumer culture are so prevalent, traditional cultural facilities serve as traditional consumption places for cultural consumption. Its attractiveness to the citizens has gradually weakened, and the cultural production space of the citizens has also turned to the cultural consumption space. Citizens' demands for cultural consumption make the space for cultural production and cultural consumption, which has been neglected in the past, receive unprecedented attention. Many cultural and creative districts, historical and cultural districts, etc. have sprung up in the city, as shown in Fig. 3.

## Gesture recognition algorithm based on feature vector

### Feature extraction

Let I be the original input image, and G is the Gaussian function. During feature extraction, $I(x,y)$ and $G(x,y,\sigma )$ need to be convolved to obtain the scale space $L(x,y,\sigma )$ of the image.

1 $$L(x,y,\sigma ) = G(x,y,\sigma )*I(x,y)$$

The Gaussian function is defined as the formula

2 $$G(x,y,\sigma ) = \frac{1}{{2\pi \sigma^{2} }}e^{{ - (x^{2} + y^{2} )/2\sigma^{2} }}$$

On this basis, $\sigma$ represents the variance of a Gaussian normal distribution, that is, the scale space factor, with large scales matching the overall characteristics of the image, and small scales corresponding to the detailed characteristics of the image^20, 21^. The characteristics of local points can be calculated by the DOG (Difference of Gaussian) operator:

3 $$D(x,y,\sigma ) = G(x,y,k\sigma )*I(x,y)$$

4 $$D(x,y,\sigma ) = L(x,y,k\sigma ) - L(x,y,\sigma )$$

Here, k is the detailed information, and its orientation is determined using the gradient directions of the neighboring pixels of the node.

5 $$m(x,y) = \sqrt {[L(x + 1,y) - L(x - 1,y)]^{2} + [L(x,y + 1) - L(x,y - 1)]^{2} }$$

6 $$\theta (x,y) = \tan^{ - 1} \frac{L(x,y + 1) - L(x,y - 1)}{{L(x + 1,y) - L(x - 1,y)}}$$

m(x,y) is the modulus of the image gradient, $\theta (x,y)$ represents the direction of the feature point, these two methods can determine the position of the feature point, and determine the scale and direction. After that, the description of the feature points is carried out^22, 23^.

### Calculation of eigenvectors

If the angle information of the joint vector of the ith frame are $\theta_{Yi}$,$\theta_{XZi}$, the calculation formulas of the motion parameter are as follows:

7 $$p_{m} = \left\{ \begin{gathered} 0,\left| {\theta_{pi} - \theta_{pj} } \right| < \Phi \hfill \\ 1,\left| {\theta_{pi} - \theta_{pj} } \right| \ge \Phi \hfill \\ \end{gathered} \right.$$

8 $$\theta_{pi} = \sum\limits_{1}^{4} {\left( {\left| {\theta_{Yi} } \right| + \left| {\theta_{XZi} } \right|} \right)}$$

9 $$\theta_{pj} = \sum\limits_{1}^{4} {\left( {\left| {\theta_{Yj} } \right| + \left| {\theta_{XZj} } \right|} \right)}$$

The experimental environment studied in this paper is immersive VR. The naive Bayes classifier can provide specific categories, and has strong robustness and computational efficiency. The following formulas are derived by using the Bayesian formula:

10 $$p\left( {y = c_{k} \left| X \right.} \right) = \frac{{\prod\nolimits_{i = 1}^{m} {w^{i} p_{x} p_{y} } }}{{\sum\nolimits_{k} {p_{y} } \prod\nolimits_{i = 1}^{m} {w^{i} p_{x} } }}$$

11 $$p_{x} = p(X^{i} \left| {y = c_{k} } \right.)$$

12 $$p_{y} = p(y = c_{k} )$$

In the formula, X represents an unclassified instance, $\{ X^{1} , \cdots ,X^{m} \}$ represents a division of X, $X^{i}$ is a characteristic attribute of X, the category set $C = \{ c_{1} ,c_{2} , \ldots ,c_{k} \}$, and $y_{i} \in C$ is a class in k classes. $w^{i} ,i = 1, \ldots ,m$ is the empirical weight, which represents the contribution of different joint vectors^24, 25^.

### Point and Hover gestures

**Figure 4:**
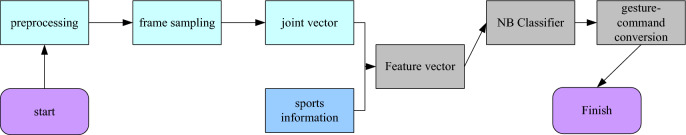
Algorithm flow.

Figure 4 shows the flow of the method. The sensor rig is initialized, the data frame is captured, the input data containing human subjects in different poses is acquired. Subsequently, the algorithm performs Pose Estimation, a crucial stage where it accurately locates key body joints or keypoints on each subject within the input data. These keypoints typically include joints like elbows, shoulders, knees, and hips, which are essential for representing the pose effectively. After Pose Estimation, the algorithm proceeds to Feature Extraction, wherein relevant information is extracted from the estimated poses. The extracted features encompass a wide range of data, such as the precise positions of keypoints, angles formed between joints, and the lengths of various limbs, all of which collectively contribute to a comprehensive representation of the pose. Armed with these features, the Pose Recognition stage begins, utilizing classification or regression algorithms to recognize and identify the specific pose displayed in the input data. The bones are sampled according to the velocity of the first step, and then the classifier is trained according to the feature vector of the first step, and then a new classifier is performed during the prediction period. The first step is to count the input training set according to formulas (7)–(11); the second step is to estimate the basic probability of each parameter $p_{x}$ and parameter $p_{y}$ with reference to the method defined in formula (12); the third step is to extract the feature vector from the input test data, and then input it into the existing classification model for classification.

## Design of urban culture based on interactive VR technology

### Simulation scene modeling technology

This section discusses the key aspects of establishing a visual simulation system, emphasizing the importance of creating a detailed three-dimensional scene model. It highlights the need for data analysis, describing elements such as brightness and structure, to ensure accurate modeling. The section also mentions the significance of real-time performance and realism in the visual system, emphasizing the requirement for fast data updates and sufficient data to create a realistic virtual environment.

When establishing a visual simulation system, establishing a three-dimensional scene model is a key part of visual roaming. Therefore, when establishing visual modeling, the data during modeling must be analyzed, and other elements in the modeling, such as the brightness and structure of the model, must be described in detail. The modeling of the three scenarios, including the design of the three scenarios and the analysis of the data, would simulate three different dimensions, and form a reasonable three-dimensional model through the unified planning and analysis of the data.

First of all, to build a three-dimensional model, there must be enough data as a basis, so that the real data can be better displayed. In this way, the real scene can be better displayed, so that the real world can be better displayed. The second problem is that in the process of constructing a 3D simulation model, it is necessary to simply simplify the real scene, so that the real scene can be restored. In this way, modeling and data display would not be affected by overly complex scenes.

For the visual system, the most important requirement is the satisfaction of the 3D scene display. The significance of the data model can be explained in two ways:

*Real-time*: Real-time refers to the speed and effect of data update, and the update speed of data is not very fast. The image of the view seen by the human eye would stay in the web of view for 0.2 s, so the speed of the system update must be within 0.2 s. In this way, the continuity of the picture can be ensured without affecting the human vision. Moreover, the drawing of the graphics is also completed within 0.2 s. That is to say, the system needs to complete the refresh and rendering of the image within 0.2 s, thereby ensuring that the video is continuous in the field of view of the human eye. The main reasons that affect the real-time performance of the visual simulation system include the detail and complexity of the scene and the organization of the scene model library, the graphics processing capability of the computer and the processing speed of computer hardware resources including the central processing, memories, unit and video memory.

*Realism*: Realism reflects a realistic VR scene. If the 3D scene is very similar to the real scene, then the realism would be higher, giving people a real feeling, and realism is a very important condition. In order to make it more realistic, a lot of data is needed to support it.

### Gesture-based immersive interaction function design

The experimental platform of this paper adopts the depth sensor and Oculus RiftDK2 to realize an immersive VR interaction system based on gestures.

Based on the depth sensing skeleton data, this paper designs an immersive virtual entity interaction system, and its hardware and software requirements are shown in Table 1.

**Table 1:** System composition.

Serial number	System composition	Development environment
1	Development Platform	Windows 10
2	Development tools	Visual Studio 2012,Unity3D 5.3.4,3ds Max 2016
3	Development language	C#, C++
4	Software platform	Kinect SDK 1.8,Oculus Runtime SDK 0.8.0.0,Leap Developer Kit 3.1.1
5	Operating environment	CPU: 3.00 GHz,GPU: 1 GB memory 810 MHz core frequency

#### Stereoscopic display

The constituent element of VR is a three-dimensional visual representation that can be continuously immersed, and to create this feeling requires the use of a 3D display, a stereoscopic display or a head-mounted display.

#### Moving track

The second technique that convinces the brain that it is in a far-off place is head motion tracking and real-time rendering. That is to say, there must be a device that allows the user to see the surrounding scene in reality.

#### Input device

To create a sense of immersion, the headset completely isolates the user's glasses from the real world, a challenge to traditional typing methods. Users must wear glasses to use the mouse and keyboard. To solve this problem, VR technology needs to develop new input devices, such as gamepads, sensors that track hand movements, and wireless body tracking devices.

#### Computer platform

Typically, VR apps are installed on the user's existing computer and smartphone, and a new desktop computer or better laptop can be connected to the Oculus Rift. All in all, the hardware and software used by the system must conform to the usage conditions of ordinary users, and at the same time ensure its functions.

**Figure 5:**
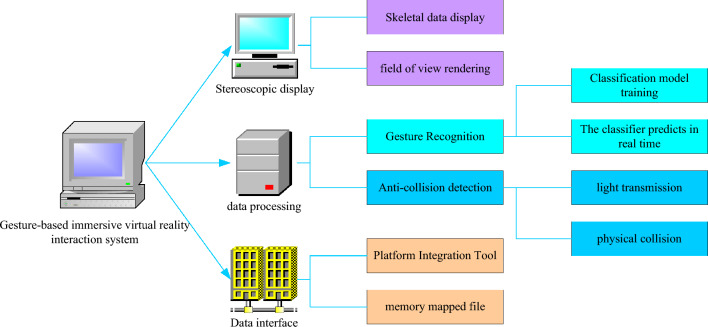
Functional block diagram.

Based on the frame structure of the gesture-based immersive VR interaction system, the main functions of the experimental platform are divided into three parts: data interface, data processing and stereoscopic display. Figure 5 shows the functional modules of the immersive VR interaction system in this paper.

The platform integration tool mainly integrates the Oculus Rift and Unity3D engines. Using the functions of the above modules, data integration is realized, which facilitates unified processing of system data. The system realizes the processing of hand spatial positioning information based on Kinect-based gesture recognition and Leap Motion tracking. The action recognition processing module further includes: a classification model training module and a real-time prediction module. The classification model training module is to perform modeling training on the extracted samples, and the real-time prediction module is to quantitatively output the classification results of the extracted samples. The collision detection module mainly detects the conflict between the hand and the interactive elements, and starts the light projection when the interactive action is found, so as to realize the selection and click of the action. If the hand collides with the interactive element directly, there would be conflict feedback based on the type of the interactive element. The stereoscopic display module is a visual way, which can visually display the interaction effect of the user, and observe and adjust the connection and operation state of the system according to the observation period provided by the system.

### System availability

#### Gesture recognition rate

**Figure 6:**
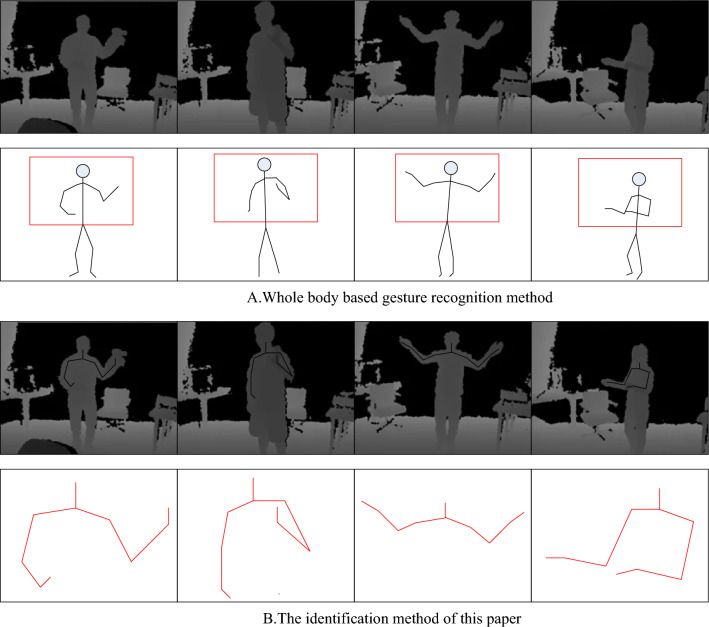
Comparison between the traditional algorithm and the gesture recognition method in this paper.

This section presents a comparison between the traditional gesture recognition algorithm and the proposed method in this paper. The traditional algorithm is to calculate the angle between different parts to get the shape feature of the hand and match it with the reference model to get the corresponding recognition effect^26^. Figure 6 is a comparison test chart between the traditional gesture recognition algorithm and the method proposed in this paper, Fig. 6A is a schematic diagram of gesture recognition of the conventional method, and Fig. 6B is a recognition schematic diagram of the algorithm here. The target's position can still be detected more accurately even when the skeletal data is unstable, and both the average and maximum recognition rates of this approach in the comparison are greater than those of the control body.

**Figure 7:**
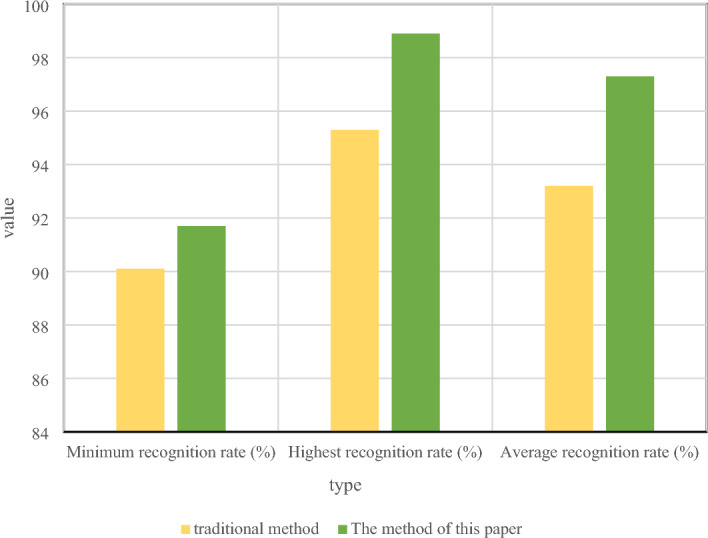
Comparison of recognition rates of the two methods.

In Fig. 6, four poses were tested, and approximately 300 samples of each pose were trained during the training phase. The results of the recognition are shown in Fig. 7 when the gestures are performed continuously, for example, if it changes from gesture 2 to gesture 1 to gesture 3, while each gesture can be recognized, it is in an immersive VR environment. When transitioning from one pose to another, if a third recognizable action occurs with an action instruction, an unexpected action by the user would occur, resulting in a wrong action. Due to the introduction of action parameters in this paper, action recognition can be performed only when the posture is stable. Therefore, if the system concludes that the posture is still changing when it is constantly changing, the action cannot be detected, helping to prevent incorrect actions. The approach used in this research has a greater recognition accuracy than the conventional way. The average recognition rate of the method in this paper is 97.3%, while the recognition rate of the traditional algorithm is 93.2%, indicating that the method in this paper has better performance.

Five gestures are chosen as the data source for comparison in order to further assess the effectiveness of the algorithm used in this study. Each gesture is merged with the joint angle and the relative head position data of the arm joint as the feature vector. In the recognition step, 2000 samples are chosen for testing, and the classification and recognition effectiveness of the algorithm is examined. The k-means classifier is used to categorize new gestures according to the Euler distance metric. The results are shown in Table 2.

**Table 2:** The recognition efficiency of the two methods.

	Traditional method	The method of this paper
Number of samples	2000	2000
Training time (ms)	183	56
Maximum recognition time (ms)	54	36
Average recognition rate (%)	98.8	97.9

As can be seen from Table 2, when the number of samples is roughly the same, it takes more time to identify the model with the traditional method, while the method proposed in this paper can quickly respond to people. Although the recognition rate of the algorithm is higher than the algorithm in this paper, on the whole, the algorithm has higher efficiency, less training time and better real-time performance.

#### Anti-collision detection

This paper takes one interface operation as an example, and conducts a series of experiments on the two interfaces respectively. As can be seen from Table 3, the first column sets the threshold value in the gesture recognition algorithm. The second column is the number of tests for each set collision detection. After repeated testing, the results show that the thresholds are $Th_{y}$ = 2.1, $Th_{z}$ = 4.5, $Th_{x}$ = 0.6, the accuracy is high, and the error can be controlled within a more reasonable range. In the experiment, the scene of menu operation is realized by laser clicking user interface options, and on this basis, collision detection based on ray projection is carried out. Experiments show that the algorithm can extract the bone type and position of the fingers according to the current gesture features, thus effectively completing the matching of gestures, and using timer and collision detection technologies to effectively identify.

**Table 3:** Collision interaction test.

Parameter settings ($Th_{y} ,Th_{z} ,Th_{x}$)	Testing frequency	Accuracy (%)
(1.0,2.2,0.3)	20	60
(2.1,4.5,0.6)	20	95
(4.2,9.0,1.2)	20	0
(2.1,0.6,4.5)	20	35
(4.5,2.1,0.6)	20	5
(4.5,0.6,2.1)	20	0
(0.6,2.1,4.5)	20	20
(0.6,4.5,2.1)	20	35

#### Interaction method usability analysis

This section explains the subjective usability testing approach and examines the findings. Natural User Interfaces (NUI) are natural, thus there aren't any specific methods to formally assess how well immersive VR works. Some people may find a design concept valuable, while others may find it difficult to employ. This implies that information may be gathered through usability tests to assess subjective comfort and usability. We created and implemented 3D interactive gestures, including a floating gesture, to test the usability of NUI. The differential algorithm is used to update the location of the angle when the virtual hand is inside the cube's impact distance, creating a more realistic pinch sensation. Dragging means that when the dummy hand comes into the slider bar collision area, the movement of the hand along the Y-axis is calculated and transferred to the slider bar, and then normalized. Thus, the slide bar's sliding position was renewed. The experiment investigated the subjective experience of the user, including the level of satisfaction and degree of usability.

Usability levels were used for all gestures, and the usability levels were divided into five measurement levels, where level 4 was an intuitive experience that did not require practice, and 0 was the worst interactive experience. The comfort level is defined by 3 criteria. Class B represents no obvious fatigue, and Class A represents a challenge to experience comfort. Table 4 lists specific categories for both assessments.

**Table 4:** Usability evaluation levels.

Availability		Comfort	
Grade	Evaluation	Grade	Evaluation
0	Not available	A	Challenging
1	Difficult to use		
2	By personal habits	B	Laborious
3	Available	C	Cozy
4	Very useful		

**Figure 8:**
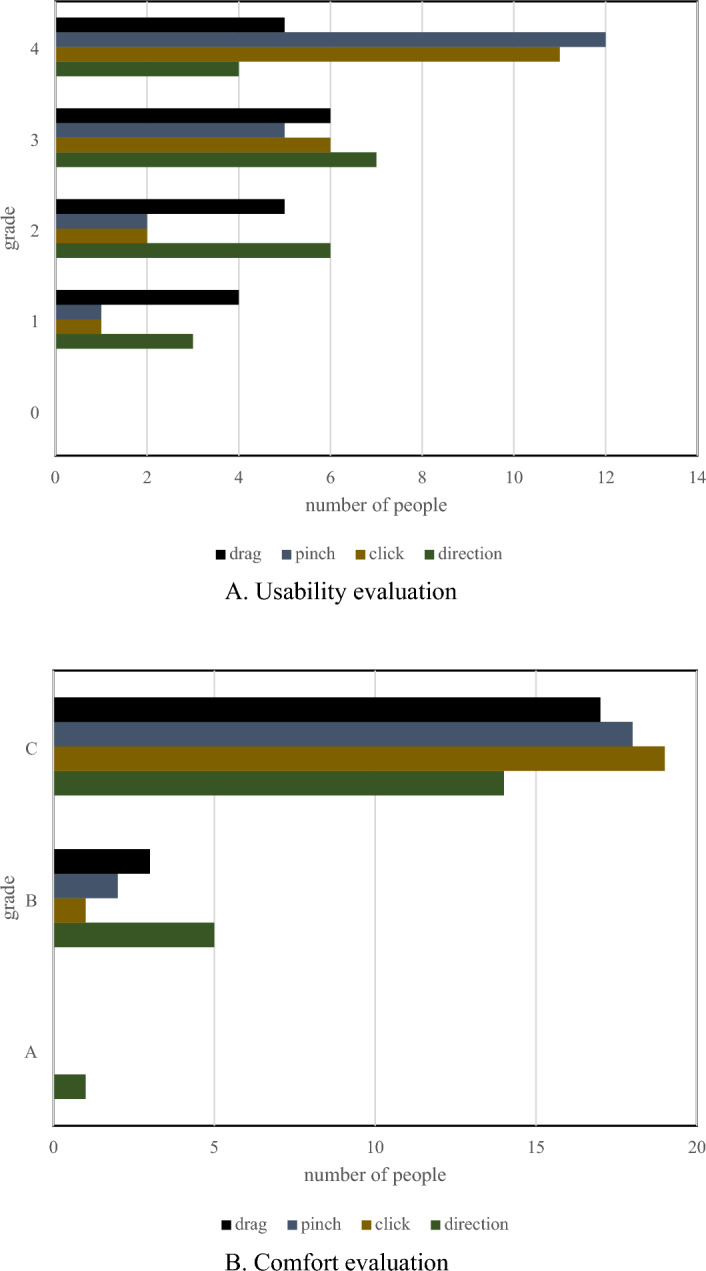
Evaluation results of the interactive method.

The experiment was conducted by testing 20 users with different perceptions of VR interaction. Figure 8 shows the evaluation of the system by different users.

In Fig. 8, the vertical axis is the evaluation level in Table 4, and the abscissa is the number of votes of users evaluating the corresponding comfort level and usability level. The interactive usability test results in DUI are shown in Fig. 8A. The interactive comfort test results in DUI are shown in Fig. 8B. In the scenario designed in this paper, the click and press gestures are closer to the user's senses and do not require any prompts. Because it aligns with real-life lifestyles, it is more comfortable to use and the user’s response is improved when guided. Both of these actions satisfy the user’s interactive needs and enhance the user's sense of immersion.

### Utilize system to design urban culture

The connotation and function of urban design is to guide and control the spatial form of the city and the strategic layout of urban activities. In terms of its characteristics, the connotation of urban design thinking is people-centered and aesthetic value as the judging standard. It comprehensively considers various social, economic, spatial and cultural elements, and finally arrives at the urban spatial form and activity strategy^27–29^. The aesthetic values it follows are not purely visual aesthetics, but should also conform to the good demands of citizens to “better city, better life”. It is oriented by spatial organization and activities to meet the needs of residents for the improvement of the living environment.

The design of urban functions with cultural themes and the exploration of cultural potential are not limited to the urbanization of urban space. Urban functions and urban space are regarded as the two joint points of cultural fusion design. Cultural concepts and traditional design are integrated into traditional design, and each stage includes the stimulation of the functional potential of cultural resources and the reflection on the spatial expression of cultural characteristics and atmosphere. It is a more in-depth cultural coupling strategy innovation.

#### Cultural implant model

The cultural implantation model is applied to new district planning, guided development, goal-oriented cultural implantation, improvement of ecological environment, enrichment of urban life, improvement of living environment, etc. Firstly, the theme culture and target culture system are established, and the urban design method is used to integrate the culture into the various urban elements of the new district, so as to achieve the purpose-centered cultural implantation. Secondly, with the support of the implementation of urban planning and the function of public policy, it would continue to regulate and guide the non-purpose culture of the new district.

#### Overall space layout

The spatial layout of the entire city includes the macroscopic texture and urban structure of the city. The urban culture carried by this type of urban space. It is the spiritual sublimation of the interaction between the city and various natural and human factors in the long historical development process, such as the imperial culture embodied by the central axis of Beijing and the regular urban pattern. For urban design, the overall spatial arrangement is the core of the entire urban planning. Under the guidance of the overall urban planning, the construction of the texture and overall structure of the city must be comprehensively considered from the aspects of historical style, cultural heritage, and natural hazards, so as to obtain the best plan.

#### Cultural carrier characteristics of urban functions

**Figure 9:**
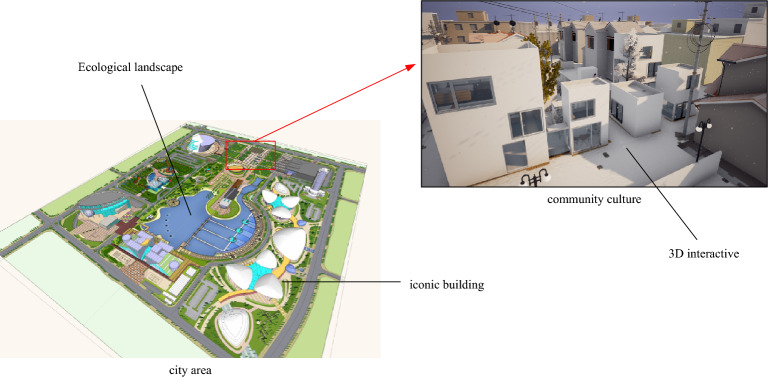
Urban cultural design concept^26^.

From the point of view of human beings, the function of a city refers to taking the city as a living environment and providing industrial and other services to residents and tourists, which are the basic attributes of the city. Urban culture is a commercial symbol, an interpretation of its economic or resource attributes. It can be used as a resource, a driving force to promote urban services, and a manifestation of culture as a functional attribute of the city. Urban life is a complex society. The interaction and enhancement of various social factors make people feel different about urban life. From the perspective of the living space provided by the city, it is a sea formed by a variety of political, economic, natural and human elements. These common perceptions and common traits appear in new forms as a new cultural connotation in the process of development. Figure 9 shows an urban cultural design rendering based on interactive VR technology.

## Conclusions

From the perspective of urban culture, urban cultural space is a place with a strong sense of civic participation, a high degree of recognizability, and a reflection of urban history and culture. This paper analyzed the formation of cultural space from three aspects: people, activities and places. It is believed that cultural space is not a purely material place, but is centered on people, and people occupy various activities in the material space, thus reflecting the culture of the city. Starting from the constituent factors and formation mechanism of cultural space, this paper summarized the basic characteristics of cultural space: participation, historical relevance, and recognizability. In order to make the research more pertinent, the spatial level of cultural space is divided: overall cultural space, cultural area and cultural facilities, with cultural area as the research object. In terms of the current situation of architecture, this paper firstly summarizes the current construction of urban cultural space in China, and points out that there is a serious urban form convergence and the lack of cultural space construction at the community level. Secondly, it summarizes the urban cultural space demands of Chinese citizens, which mainly include three levels: pride in the city, self-identification, and emphasis on cultural industries. The conflict between this demand and the status quo of the building illustrates the strengthening of the construction of cultural spaces (especially cultural industrial spaces) at the community level, while at the same time reflecting its unique regional characteristics.

## Data Availability

The experimental data used to support the findings of this work are available from the corresponding author upon request.
